# Exploring Predictors of Social Media Use for Health and Wellness during COVID-19 among Adults in the US: A Social Cognitive Theory Application

**DOI:** 10.3390/healthcare12010039

**Published:** 2023-12-23

**Authors:** Safa Elkefi

**Affiliations:** 1 School of Nursing, Columbia University, New York, NY 10032, USA; hphactors@gmail.com; Tel.: +1-(201)-744-1208; 2 Hphactors Lab, New York, NY 10022, USA

**Keywords:** social media, social isolation, purpose and meaning in life, social cognitive theory, self-efficacy, health perception, COVID-19

## Abstract

During COVID-19, SM media was relied upon for health-related information-seeking and activity support. This study uses the social cognitive theory (SCT) and a representative dataset of the population in the US to explore the factors influencing patients’ perceptions of SM for health-related activities. As per SCT, consolidated factors comprised personal factors (sociodemographic, health perception, self-efficacy) and environmental factors (social isolation, purpose in life). Multivariate logistic regression analysis was conducted. Among the 6252 respondents, 95.15% rarely use SM to share personal health-related information, and 90.44% rarely use it to share general health-related information. Older individuals and Whites are less likely to consider SM for healthcare decisions. Education levels influence SM’s perceived reliability. Those with positive health perceptions find SM more suitable for healthcare discussions. Socially-isolated individuals are less likely to use SM for healthcare. Those with a strong sense of purpose are less inclined to trust it for health decisions and may question its accuracy. SM-based interventions should address sociodemographic differences. Our findings contribute to the literature by SCT relevance validation in identifying the antecedents of SM use in healthcare. Our results also help to understand the challenges to its adoption. This can help enhance SM-based communication strategies and interventions.

## 1. Introduction

### 1.1. Background

There is an ongoing increase in the use of social media (SM) globally [1], including in healthcare contexts by various users [1,2,3,4]. Based on a study published in 2017, 70% of Americans currently use social media to connect, which can be compared with only 5% in 2005 [5]. In 2019, almost 9 out of 10 adults in the USA used at least one social media network [6]. In 2020, there were over 4.74 billion social media users worldwide, equating to 59.3 percent of the global population [7].

Many studies have shown that the introduction of SM-based communication changed the nature and speed of interactions between different actors in healthcare [4]. For example, networks and platforms such as Facebook, Twitter, Blogs, YouTube, and Yelp have helped accelerate healthcare’s democratization through enabling patients to take a much more active and informed role in managing their health [6]. Due to the inherent social nature of SM, it is becoming one of the most preferred venues for obtaining health information and community support [8]. These usages have been shown to influence health behavior [9]. Studies supporting SM use have shown it offers opportunities for health improvement as it enhances connection with peers, improves the efficacy of technology-based health interventions, and provides more equitable access to health information to different patients [10,11,12,13,14]. In addition, SM has been considered by healthcare workers to be a communication system through which they can find patients’ health information and past medical history [15,16]. It has also helped them overcome the barriers to healthcare delivery for patients [3]. 

Despite its benefits, some studies have identified potential concerns related to the use of SM in healthcare, such as the accuracy of the information, quality issues, potential information overload, and data security issues [1]. Increased use of SM has been associated with mental health challenges among young adults and adolescents and harmful behavior promotion, such as of violence and addiction [17,18,19]. In addition, the capacity of SM to spread health misinformation has been apparent through the falsified COVID-19-related news and rumors circulating on different platforms [20]. For patients who may lack health and media literacy skills, critically evaluating the information encountered on SM platforms may be challenging. As individuals turn to online sources for answers, they may encounter an “infodemic”, making it hard to find reliable sources and accurate information when needed [7,21]. Thus, it remains important to generate up-to-date examinations of its role. Furthermore, as more and more interventions resort to SM for better patient accessibility, more control over the possible factors that can impact the success and failure of such interventions should be considered [22]. To date, no study has investigated the predictors of adults’ decisions to use SM for health-related purposes. This study aims to (1) explore the use of SM for health endeavors among different groups and to (2) determine the potential factors influencing patients’ perceptions of social media use for different health-related activities using nationally representative data following the social cognitive theory (SCT) framework. 

### 1.2. Theoretical Background

Bandura’s SCT is rooted in the psychology of human learning and behavior [23,24]. It advocates a long-term framework for influencing and motivating human behavior, linking theoretical stances to health education and promotion paradigms [23,24]. This study postulates that environmental and personal factors influence the use of social media for health-related endeavors. Appendix A summarizes all the variables and questions used. The utilization of SCT within this study serves as a foundational framework for the analytical investigations conducted. As elucidated in the theoretical background, SCT is instrumental in guiding the formulation of hypotheses through facilitating the identification of antecedents. The adoption of SCT in this context is driven by its efficacy in providing a structured lens to examine and understand the factors influencing health information behavior. It is essential to underscore that the application of SCT is not necessarily prescriptive but rather serves as a methodological tool to enhance the scientific rigor of the study. The intention is not to advocate for a specific behavior, such as utilizing social media for health information, but to leverage SCT as a theoretical scaffold for a nuanced exploration of the interplay between variables and their impact on health information-seeking behaviors.

#### 1.2.1. Personal Factors

##### Demographics

Research has shown a relationship between personality traits and engagement with social media [25]. Age, gender, education level, and other factors impact internet access among different populations [25,26]. Thus, we hypothesize that demographics (age, gender, race, education, and income) predict different SM use perceptions (Hypothesis 1).

##### Health Perception

Many studies have linked health perceptions to social media use [27]. Buda and colleagues showed that problematic social media use among teenagers is associated with about twice the odds of worse sleep quality and lower life satisfaction [28]. Their study adds to the literature showing that social media use among teenagers may cause negative health perceptions and outcomes [28]. Thus, we hypothesize that patients’ health perception is associated with using SM for health-related purposes (Hypothesis 2). 

##### Self-Efficacy

Finally, self-efficacy is a proximal and direct predictor of health behavior [29,30]. According to social cognitive theory, a personal sense of control facilitates health behavior change [31]. Thus, we hypothesize that self-efficacy is associated with using SM for health-related purposes (Hypothesis 3).

#### 1.2.2. Environmental Factors

Environmental factors in SCT refer to the physical, social, and cultural context in which individuals are situated [23,24]. These factors can influence behavior through the process of social modeling. Individuals are more likely to adopt behaviors they see modeled by others in their environment [23,24]. For example, if an individual sees their peers engaging in risky behavior, they may be more likely to do so themselves [32]. Research has also shown that social support can be associated with health-related behavior [33]. We hypothesize that social isolation and meaning and purpose in life impact the use of SM for health-related purposes (Hypothesis 4 and Hypothesis 5). 

To conclude, SCT is important in understanding the use of SM for health-related purposes. Our study aims to investigate the use of SM for health purposes among different population groups. It also explores the factors that may impact the patient’s use of SM for health-related purposes following SCT. 

This section presents the hypotheses that we will test:

**Hypothesis 1.**  *Demographic factors are associated with SM use for health purposes.*

**Hypothesis 2.**  *Health perception is associated with SM use for health purposes.*

**Hypothesis 3.**  *Self-efficacy is associated with SM use for health purposes.*

**Hypothesis 4.**  *Social Isolation is associated with SM use for health purposes.*

**Hypothesis 5.**  *Meaning and Purpose in life is associated with SM use for health purposes.*

## 2. Materials and Methods

This study is a secondary data analysis. This section will present the data and the analysis methods to address the hypotheses.

### 2.1. Data Sources and Sampling Methodology 

We used data from cycle 6 of the Health Information National Trends Survey (HINTS) collected by the National Institute of Health [34]. HINTS is a nationally representative cross-sectional survey of non-institutionalized adults (18 years or older). Data collection for HINTS 6 occurred when COVID-19. It started on 7 March 2022, and concluded on 8 November 2022 [35]. The data collected were related to the year of 2021 when COVID-19 was at its pick in the US hitting a weekly death rate of 21,332 in January 2022 and of 25,974 in January 2021 based on CDC statistics [36]. 

The sampling strategy for the HINTS 6 survey consisted of a two-stage design [35]. In the first stage, a stratified sample of addresses was selected from a file of residential addresses. In the second stage, one adult was selected within each sampled household [35]. The sampling frame for HINTS 6 consisted of a database of addresses used by Marketing Systems Group (MSG) to provide random samples of addresses. The sample was stratified according to breakouts of minority and rural status [35]. The purpose of creating sampling strata by breakouts of minority and rural status was to provide a means to sample the high-minority and rural strata at higher rates relative to the low-minority and urban strata to increase the precision of estimates for minority and rural populations [35]. 

Respondents to HINTS 6 were allowed to complete the survey online or on paper [35]. Both modes of the survey (paper and online) were offered in English or Spanish [35]. All participants received a $2 pre-paid monetary incentive to encourage survey completion [35]. Overall, 6505 responses were received. Returned surveys were reviewed for completion and duplication (more than one questionnaire returned from the same household) to ensure they were eligible for inclusion in the final dataset. Of the 6505 questionnaires received, 27 were returned blank, 148 were determined to be incompletely filled out, and 78 surveys were identified as duplicates (i.e., the same household returned multiple surveys). The remaining 6252 surveys were determined to be eligible [35]. Further details on study design, survey design, data collection, and cleaning can be found on the HINTS website [37] and in previous studies [38].

### 2.2. Measures and Instrumentation 

**Figure 1:**
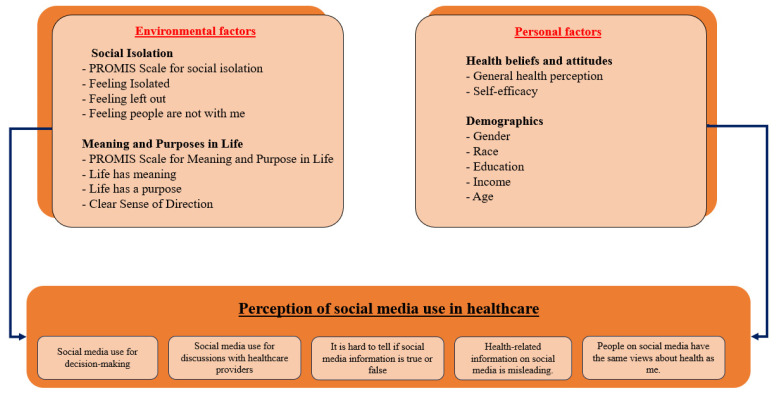
Conceptual framework of the study.

All the questions and variables used in this study are summarized in the Appendix A. Figure 1 summarizes the factors used:

This study has two main parts of analysis. The first part explores how often the respondents used SM during the 12 months preceding data collection. For that, the variables used are: (1) visiting SM, (2) using SM to share personal health information, (3) using SM to share general health information, (4) using SM to interact with people with similar health issues, and (5) using SM to watch health-related videos. These questions were used to explore the overall use trends among different populations. The questions were categorized into three levels: daily use, sometimes a month, and less than once a month.

The perception of respondents towards SM-related information is considered in the second part. Five variables were considered in testing whether patients agree or not about (1) using SM information to make decisions about their health, (2) using SM information in discussions with their healthcare providers, (3) finding it hard to tell if SM information is true or false, (4) people on SM having the same views about health as them, and (5) health-related information on SM being misleading. For these five questions, response options were “strongly agree”, “agree”, “somewhat disagree”, and “strongly disagree.” The responses were dichotomized into “agree” and “disagree” for analysis. The categorization of the variables is supported by previous studies using the same dataset [39]. 

#### Independent Variables: Personal and Environmental Factors

The personal factors considered include health-related perceptions and demographic variables. The demographic variables considered are age, race, gender, income, and education. For health-related perceptions, the variables considered are general health perception and self-efficacy in managing one’s health. 

The environmental factors are quantified by measure from the Patient-Reported Outcomes Measurement Information System (PROMIS) [40]. PROMIS is a set of measures used to evaluate physical, social, and mental health [40]. PROMIS was initiated in 2004 and funded by the United States National Institutes of Health [41]. This initiative was led by Northwestern University, in partnership with six other American academic institutions, to build and validate a common, accessible item bank to measure key symptoms and health domains applicable to a range of chronic conditions [41]. The PROMIS measure’s scales used in this study are social Isolation [42] and meaning and purpose in life and their components [43]. We considered the social isolation score and its components (I feel isolated, I feel left out, I feel that people barely know me, and I feel that people are not with me) and purpose and meaning in life score and its components (life has meaning, life has a purpose, and I have a clear sense of direction). The questions have five categories: “never”, “rarely”, “sometimes”, “usually”, and “always.” To facilitate the interpretation, the answers are categorized into three levels of: “yes”, “sometimes”, and “no.” 

### 2.3. Statistical Analyses 

For the descriptive statistics, the proportions of respondents who reported their use of social media overall and of different demographic groups are calculated. Wald chi-square tests are used to determine the association between the different usages and the characteristics of the respondents. A *p*-value < 0.05 is considered significant. Then, multivariate logistic regression models examine the relationships between the dependent and independent variables outlined above. The model-building process involved three steps for each of the five dependent variables. The dependent variables are dichotomized for analysis purposes [30,44]. Odds ratios (OR) are presented with 95% confidence intervals (CI) and *p*-values for the final adjusted models, are presented with statistical significance set at *p* < 0.05. To handle missing data, a multiple imputation method is used. Multiple imputations are a useful strategy for handling missing data problems and accounting for the uncertainty of imputation [45,46]. The k-nearest-neighbour algorithm method is used when the missing values get replaced by nearest neighbor estimated values [46]. Analyses were performed using Python software, version 3.8 (Python, Hoboken, NJ, USA), using complex survey design procedures (researchpy, numpy, pandas, statsmodels, sklearn, etc.). 

### 2.4. The Characteristics of the Study’s Participants

The pooled data included 6252 respondents from the US. The sample’s characteristics are detailed in Table 1. Approximately two-thirds of the respondents were older than 50. A majority of 61.44% of the respondents were females. A total of 16.76% of the respondents were African Americans and 53.58% were white respondents. A rate of 46.10% of the respondents were college graduates, and 35.27% had an income higher than 75 thousand dollars. Most respondents (82.53%) thought they had good health and (71.88%) could care for their health. 

## 3. Results

### 3.1. Use of Social Media across the Different Demographic Groups

**Figure 2:**
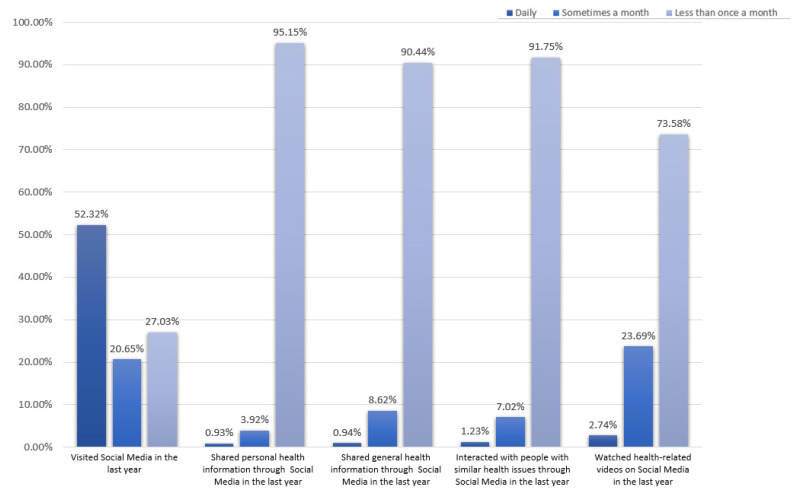
Use of social media for health-related activities.

Despite 52.32% of the respondents accessing SM every day and only 27.03% accessing it less than once a month, most do not use it regularly to access health-related information, as shown in Figure 2. A total of 95.15% of the respondents said that they rarely use SM to share personal health-related information, and 90.44% mentioned rarely using it to share general health-related information. In addition, less than 7.02% use SM to interact frequently with other people with similar health issues, and only 2.74% use it frequently to watch health videos online.

As shown in Table 2, accessing social media is associated with the characteristics of the respondents. For instance, visiting social media correlated with age, gender, income, and education level. Young people tend to access social media more frequently than older people. A total of 81.36% of young respondents between 18 and 34 said they visit social media platforms daily versus 4.26% of them accessing social media platforms once a month or less. However, 46.55% of people over 64 access it only once a month or less. Females access social media more than men (daily: 55.32% females vs 47.53% males and once a month or less: 24% female vs 31.85 males). People with higher income (75k or more) access SM daily (60.14%). Despite these trends, the use of SM for health-related purposes depends on the population’s demographic characteristics but remains low. Only 1.92% and 2.66% of the young respondents mentioned accessing SM daily to share personal health information and interact with a healthcare provider. Respectively, 89.46% and 84.56% of them accessed it less than once a month. This is also consistent with the older population (65 or older), as 97.22% used SM to share personal health information less than once a month. 

Finally, more people use SM to watch health-related videos. A total of 46.32% of the young population mentioned accessing SM for that purpose at least a few times a month, which is still low compared with older adults (15.11%). This usage is also lower among white people and people with higher incomes than others. The higher the income, the less people rely on SM to share and access health information, interact with healthcare providers, and watch health-related videos.

### 3.2. Perception of Social Media Use for Health-Related Purposes

Age strongly predicts the respondents’ perceptions towards SM use for health-related purposes. The older populations are, the less likely they are to believe they should use SM to make decisions about their health or in discussions with their healthcare providers (HCP) than young patients (for decision making at 65 years or more: OR = 0.52, *p* < 0.001; at 50 yrs-64: OR = 0.7, *p* = 0.002). They also are less likely to think people on SM have the same views about their health as them (OR = 0.61, *p* < 0.001). Older people find it harder to tell whether health information on SM is true or false (OR = 2.36, *p* < 0.001). They are less likely to think that health information on SM is misleading (OR = 0.11, *p* < 0.001). 

In addition, white populations are less likely to rely on SM health information to make health decisions than other populations (OR black = 1.57, *p* < 0.01; OR hispanic =1.35, *p* = 0.004). Furthermore, education level predicts the respondent’s perception of the reliability of SM. The higher the educational level respondents have, the more likely they are to think that it is hard to tell whether health information on social media is true or false (OR = 2.98, *p* < 0.001) and to think that a lot of the information on SM is misleading for them (OR = 3.27, *p* < 0.001). Moreover, people who think their health is good are more likely to think they can use SM in discussions with their healthcare providers than people who think their health status is bad (OR = 1.26, *p* = 0.04). People’s perspective about using SM for healthcare purposes also depends on their social situation. For instance, people who are socially isolated are more likely to not use SM in discussions with their HCP, to think that people on SM do not have the same views about health as them, and to think that information on SM is misleading or false (respectively, OR = 1.08, *p* = 0.009; OR = 2.81, *p* = 0.01; OR = 3.36, *p* = 0.005). Finally, people who have meaning and purpose to their lives are less likely to think that they can use SM to make decisions about their health (OR = 0.89, *p* = 0.048) and are more likely to be confused about whether health information on SM is true or false (OR = 1.95, *p* = 0.006).

## 4. Discussion

The paper presents the findings on using social media (SM) for health-related purposes based on a survey of 6252 respondents from a nationally representative database. The results show that although most respondents accessed SM daily, they rarely use it for health-related purposes, which align with previous studies’ results [47,48]. 

### 4.1. Demographics

SM usage was influenced by socioeconomic factors (gender, income, education level, and race). Younger people accessed SM more frequently than older people. The relatively low penetration of SM use among older adults is consistent with other studies’ findings [49]. This finding suggests that using SM for communication may not be at its peak for all populations, which may raise health disparities among this age group. Additionally, female respondents were also found to access SM more than male respondents, and those with higher income were more likely to access it daily. However, despite these trends, the use of SM for health-related purposes remained low across all demographic groups. The paper also found that older populations were less likely than young patients to believe that they should use SM to make decisions about their health or in discussions with their healthcare providers (HCP). They were also less likely to think that people on SM had the same views about their health as them and found it harder to tell whether health information on SM was true or false. In contrast, younger people were likelier to access SM to share personal health information and interact with a healthcare provider. 

Based on this study’s findings, higher levels of education predict a more skeptical attitude toward the reliability of health information on social media, which is not surprising. It is well-established that higher education is associated with a stronger critical thinking ability, which allows individuals to evaluate the credibility of the information they come across [50,51,52,53]. The demographic variations in social media (SM) usage suggests that health communication strategies need to be tailored to specific groups. Different age groups, genders, income levels, and education backgrounds may require different approaches to effectively communicate health information. Furthermore, efforts should be made to bridge the digital divide among older populations to ensure they have access to reliable health information through social media. Despite the overall trends in social media use, the finding that its use for health-related purposes is low across all demographic groups emphasizes the need for targeted public health campaigns. Strategies should be developed to promote using social media as a reliable source of health information. Moreover, younger people being more likely to share personal health information on social media suggests the importance of promoting responsible sharing practices. Individuals should be educated on the potential consequences of sharing health information online and encouraged to engage in meaningful discussions with healthcare providers instead.

### 4.2. Health Perception

This study also showed that people with good health perceptions are more likely to use SM in discussions with their healthcare providers. It is widely recognized that an individual’s perception of their health can influence various aspects of their behavior [54,55]. Individuals who perceive their health as good often exhibit higher confidence levels in managing their health and seeking information about their health [56]. This confidence may extend to their belief in using SM as a valuable tool for engaging in their health experience, as found in this study. Individuals who perceive their health as poor may approach SM interactions more cautiously or skeptically. These findings have different implications. For instance, tailoring messages to resonate with individuals’ positive health perceptions can enhance engagement and encourage healthier behaviors. Promoting health confidence through educational initiatives and support systems can empower individuals to manage their health actively. Social media can be a platform to reinforce positive health behaviors and provide relevant information. Healthcare organizations may consider incorporating social media into their communication and engagement strategies. This could involve creating official social media channels for health-related information, hosting discussion sessions, and providing a platform for patients to share positive health experiences.

### 4.3. Social Isolation

Although SM can be an alternative interaction source for people with social isolation issues, this study found that socially isolated individuals were less likely to use SM for health-related purposes. Socially isolated individuals were likelier to think that information on SM was misleading or false. This study’s findings correlate with other studies suggesting that socially isolated adults are more likely to have negative perceptions and problems using SM [57,58]. Different reasons can explain this perception. For instance, socially isolated people have limited contact with others, making them seek alternative means of interaction, support, and information to substitute for their in-person interactions. SM platforms may provide a virtual space that can allow them to maintain social connectedness [59]. However, this does not guarantee their trust in SM as a source of information. Recognizing that socially isolated individuals are less likely to use social media for health-related purposes suggests the need for tailored health communication strategies. Healthcare providers and public health campaigns should explore alternative channels to reach socially isolated individuals, ensuring they receive accurate and relevant health information. In addition, establishing official and verified health-related accounts on SM platforms may help build trust among socially isolated individuals. Furthermore, SM platforms can design features or interventions to foster community and trust among socially isolated users.

### 4.4. Sense of Meaning and Purpose in Life

Finally, this study consolidates the relationship between individuals with a sense of meaning and purpose in life and their attitudes towards using SM for health purposes. The findings revealed that individuals with a strong sense of meaning and purpose were less likely to consider SM a reliable platform for making decisions about their health. Additionally, they were more likely to experience confusion regarding the integrity of health information found on SM. This outcome underscores the importance of psychological strengths, such as having meaning and direction in one’s life that impacts one’s behavior, as shown in other studies [60]. Many studies show that having a high sense of purpose correlates with less unhealthy behavior [61]. Patients may consider using SM for health purposes an unhealthy behavior, which may explain this result. Participative SM has brought rapid changes to the landscape of communication. Thus, it is necessary to develop a better understanding of these technologies and how they may impact health communication among different populations. To effectively engage different demographic groups, targeted SM interventions should account for sociodemographic differences to address their specific needs. Health communication strategies should also be adaptive, evolving with technological changes and societal perceptions to engage different demographic groups effectively. Further research into the reasons behind this perception can provide insights into developing interventions that promote positive health behavior while addressing individual concerns.

### 4.5. Study Contribution

This study delves into the nuanced relationship between social media (SM) use for health-related purposes and various factors, drawing insights from a survey of 6252 respondents. The findings reveal that while daily SM access is common, its utilization for health-related reasons remains low across demographic groups, echoing previous studies. Demographic factors such as age, gender, income, and education influence SM usage, highlighting the need for tailored health communication strategies. The study emphasizes the importance of addressing the digital divide among older populations to ensure equitable access to reliable health information. Furthermore, the research highlights the significance of health perceptions, social isolation, and a sense of meaning and purpose in shaping attitudes towards SM use for health. Tailoring messages to individuals’ positive health perceptions, targeting socially isolated individuals with alternative communication channels, and understanding the impact of a sense of purpose on SM perceptions are vital for effective health communication strategies. Overall, the study provides valuable insights into developing targeted public health campaigns and interventions to promote responsible SM use and engage diverse demographic groups in health-related discussions.

### 4.6. Study Limitations

Our results provide an important starting point for future studies on SM use for health-related purposes. However, this study has some limitations. First, the cross-sectional survey may prevent us from drawing accurate causal inferences about the observed relationship between variables. Second, respondents self-report sociodemographic variables, SM use, and other behaviors which may not be accurate and depend on the recall and the correct interpretation of survey questions and response options. Finally, the survey questions related to SM use are limited to specific usages. Thus, the data does not provide a comprehensive generalizable picture of SM use for health-related purposes. For instance, individuals might be turning to SM to find tips and motivation for achieving health goals such as weight loss, this health-related use is not captured in the current iteration of the survey. Despite these limitations, this study provides an important and timely update regarding SM use generally and for health-related purposes among adults and suggests important implications for health communication research and practice.

## 5. Conclusions

Based on SCT, this study investigates the use of SM for health endeavors and the factors influencing it. We found that a low number of participants used SM to share general or personal health information. Education, race, and age correlate with this behavior. People with positive health perceptions find SM more suitable for healthcare discussions. Socially isolated individuals tend not to use SM for health-related purposes, and those with a strong sense of purpose are less inclined to trust SM for health decisions and may question the accuracy of health information. SM interventions should account for sociodemographic differences to prevent misinformation. To promote engagement in health interventions using SM, it remains important to understand the social barriers and challenges faced by individuals accessing it. Finally, further understanding the psychological factors that influence people’s attitudes towards SM use for health-related purposes can help improve and tailor communication strategies and interventions.

## Figures and Tables

**Table 1:** Characteristics of the sample.

Variable		N	%
Age	18–34	939	15.02%
	35–49	1249	19.98%
	50–64	1834	29.33%
	65 or more	2230	35.67%
Gender	Male	2411	38.56%
	Female	3841	61.44%
Race	White	3350	53.58%
	Black	1048	16.76%
	Hispanic	1382	22.10%
	Other	472	7.55%
Education	Less than high school	387	6.19%
	High school graduate	1099	17.58%
	Some college	1884	30.13%
	College graduate or more	2882	46.10%
Income	Less than 20k	969	15.50%
	20k to 35k	837	13.39%
	35k to 50k	998	15.96%
	50k to 75k	1243	19.88%
	75k or more	2205	35.27%
WorkFullTime	No	3218	51.47%
	Yes	3034	48.53%
GeneralHealth	Bad	156	2.50%
	Fair	936	14.97%
	Good	5160	82.53%
Self-efficacy	Bad	337	5.39%
	Somehow	1421	22.73%
	Good	4494	71.88%

**Table 2:** Results of the logistic regression models testing the association between the predictors and the use of Social Media for health and wellness.

			SocMed MakeDecisions		SocMed DiscussHCP		SocMed TrueFalse		SocMed SameViews		Misleading Health Information	
			*p*	OR	*p*	OR	*p*	OR	*p*	OR	*p*	OR
*Personal Factors*												
Demographics	Age	18–34										
		35–49	0.406	0.9 [0.72–1.14]	0.015 *	0.76 [0.62–0.95]	0.218	1.33 [0.94–1.12]	<0.001 ***	0.8 [0.56–0.67]	<0.001 ***	0.68 [0.29–0.44]
		50–64	0.002 **	0.7 [0.56–0.88]	0.003 **	0.73 [0.59–0.9]	<0.001 ***	1.54 [1.09–1.3]	<0.001 ***	0.76 [0.54–0.64]	<0.001 ***	0.23 [0.11–0.16]
		65 or more	<0.001 ***	0.52 [0.4–0.67]	<0.001 ***	0.63 [0.5–0.79]	<0.001 ***	2.36 [1.62–1.95]	<0.001 ***	0.61 [0.42–0.5]	<0.001 ***	0.11 [0.05–0.08]
	Gender	Male										
		Female	0.355	1.08 [0.92–1.27]	0.052	1.16 [1.01–1.35]	0.856	1.11 [0.88–0.99]	0.194	1.21 [0.96–1.08]	0.146	1.64 [1.25–1.43]
	Race	White										
		Black	<0.001 ***	1.57 [1.27–1.93]	0.001	1.38 [1.14–1.68]	0.170	0.88 [0.65–0.76]	0.071	1.01 [0.74–0.86]	0.063	1.29 [0.86–1.05]
		Hispanic	0.004 **	1.35 [1.1–1.65]	0.205	1.13 [0.94–1.36]	0.250	0.97 [0.73–0.84]	<0.001 ***	0.87 [0.65–0.75]	0.460	1.11 [0.79–0.94]
		Other	<0.001 ***	1.8 [1.39–2.35]	0.001	1.52 [1.2–1.95]	0.643	1.17 [0.77–0.95]	0.125	1.05 [0.69–0.85]	0.440	1.51 [0.84–1.12]
	Education	<High School										
		High school graduate	0.105	0.75 [0.53–1.06]	0.165	0.79 [0.56–1.1]	<0.001 ***	1.99 [1.22–1.55]	0.836	1.36 [0.78–1.03]	0.012 *	1.61 [0.95–1.23]
		Some college	0.607	0.91 [0.66–1.28]	0.753	1.05 [0.76–1.45]	<0.001 ***	2.32 [1.44–1.82]	0.95	1.3 [0.76–0.99]	<0.001 ***	3.04 [1.79–2.34]
		College Graduate	0.668	1.07 [0.77–1.51]	0.267	1.2 [0.87–1.67]	<0.001 ***	2.98 [1.21–4.55]	0.263	1.54 [0.89–1.17]	<0.001 ***	3.27 [1.89–2.48]
	Income Levels	Less than 20K										
		20k to 35k	0.396	0.89 [0.67–1.17]	0.950	0.8 [0.61–1.04]	0.111	1.44 [0.96–1.17]	0.809	1.28 [0.83–1.03]	0.069	1.54 [0.98–1.23]
		35k to 50k	0.474	0.9 [0.69–1.19]	0.200	0.73 [0.57–0.95]	0.480	1.49 [1–1.22]	0.524	1.33 [0.87–1.07]	0.070	1.68 [1.07–1.34]
		50k to 75k	0.411	0.89 [0.68–1.17]	0.246	0.86 [0.67–1.11]	0.220	1.76 [1.18–1.43]	0.170	1.59 [1.05–1.28]	0.082	2.17 [1.36–1.72]
		75k or more	0.290	0.74 [0.56–0.97]	0.36	0.77 [0.6–0.98]	0.670	1.67 [1.13–1.36]	0.190	1.57 [1.04–1.28]	0.095	2.5 [1.57–1.97]
Health-Beliefs & Attitudes	General Health	Bad										
		Fair	0.886	0.96 [0.57–1.63]	0.064	1.13 [0.67–1.9]	0.406	1.25 [0.58–0.85]	0.550	1.33 [0.58–0.88]	0.413	1.29 [0.54–0.84]
		Good	0.913	0.97 [0.57–1.65]	0.04 *	1.26 [0.75–2.12]	0.838	1.53 [0.71–1.04]	0.569	1.7 [0.75–1.13]	0.620	1.39 [0.58–0.9]
	Self Efficacy	Bad										
		Somehow	0.852	0.96 [0.66–1.4]	0.614	1.11 [0.76–1.59]	0.940	1.31 [0.75–0.99]	0.261	1.45 [0.8–1.08]	0.363	1.64 [0.83–1.17]
		Good	0.450	1.01 [0.69–1.48]	0.013	1.32 [0.92–1.89]	0.227	1.26 [0.71–0.95]	0.352	1.57 [0.85–1.16]	0.722	1.51 [0.75–1.06]
*Environmental Factors (Psycho-social)*												
Social Isolation	Feeling Isolated	No										
		Somehow	0.553	1.13 [0.75–1.69]	0.039 *	0.97 [0.77–1.22]	0.026 *	1.08 [0.74–0.9]	0.012 *	1.53 [1.06–1.27]	0.006 **	1.8 [1.1–1.4]
		Yes	0.010 *	1.39 [1.08–1.78]	0.041 *	0.85 [0.59–1.24]	0.032 *	1.16 [0.63–0.85]	0.036 *	1.59 [0.84–1.16]	0.006 **	1.68 [0.73–1.12]
	Feeling left out	No										
		Somehow	0.094	0.99 [0.66–1.48]	0.015	1.31 [0.9–1.91]	0.042 *	1.31 [0.93–1.11]	0.039 *	1.28 [0.91–1.07]	0.892	1.26 [0.82–1.01]
		Yes	0.017 *	1.17 [0.93–1.48]	0.019 *	1.15 [0.93–1.41]	0.025 *	1.44 [0.77–1.05]	0.074	1.45 [0.77–1.05]	0.528	1.34 [0.57–0.87]
	Feeling People Barely Know Me	No										
		Somehow	0.131	1.31 [0.92–1.85]	0.036	1.4 [1.02–1.93]	0.354	1.28 [0.92–1.08]	0.650	1.14 [0.82–0.96]	0.074	2 [0.97–1.39]
		Yes	0.033 *	1.27 [1.02–1.6]	0.014 *	1.28 [1.05–1.58]	0.662	1.23 [0.72–0.94]	0.447	1.18 [0.69–0.9]	0.033 *	1.56 [1.02–1.26]
	Feeling People Not With Me	No										
		Somehow	0.087	1.97 [1.67–3.41]	0.061	0.94 [0.75–1.18]	0.069	1.25 [0.86–1.04]	0.053	1.13 [0.78–0.94]	0.305	1.12 [0.69–0.88]
		Yes	0.027 *	1.75 [1.58–2.97]	0.039 *	0.86 [0.6–1.22]	0.078	1.28 [0.72–0.96]	0.028 *	1.14 [0.63–0.85]	0.625	1.34 [0.62–0.9]
	PROMIS Social Isolation	No										
		Somehow	0.090	1.23 [0.75–1.51]	0.005 **	1.31 [1.08–1.59]	0.449	1.22 [0.92–1.05]	0.001 **	1.46 [1.1–1.26]	0.003 **	1.42 [1.02–1.2]
		Yes	0.088	1.38 [0.74–2.54]	0.009 **	1.08 [1.12–1.92]	0.407	1.95 [0.76–1.22]	0.010 *	2.82 [1.11–1.79]	0.005 **	3.36 [0.95–1.79]
Purpose and Meaning in Life	Life Has Meaning	No										
		Somehow	0.075	0.9 [0.51–1.58]	0.092	1.02 [0.64–1.65]	0.009 **	2.47 [1.14–1.68]	0.014 *	2.00 [0.91–1.35]	0.716	1.82 [0.66–1.09]
		Yes	0.046 *	0.81 [0.48–1.37]	0.065	0.89 [0.53–1.49]	0.010 *	2.14 [0.93–1.4]	0.009 **	2.22 [0.93–1.43]	0.166	2.52 [0.85–1.46]
	Life Has Purpose	No										
		Somehow	0.013 *	1.51 [0.91–2.51]	0.038 *	1.22 [0.78–1.92]	0.064	1.57 [0.76–1.09]	0.229	1.15 [0.56–0.8]	0.014 *	0.89 [0.34–0.54]
		Yes	0.023 *	1.43 [0.83–2.46]	0.019 *	1.39 [0.85–2.27]	0.074	1.39 [0.63–0.93]	0.227	1.17 [0.52–0.78]	0.016 *	1.11 [0.39–0.66]
	Clear Sense of Direction	No										
		Somehow	0.020 *	0.72 [0.55–0.95]	0.046 *	0.91 [0.72–1.16]	0.076	1.18 [0.8–0.97]	0.061	1.47 [0.99–1.21]	0.022 *	1.53 [0.9–1.17]
		Yes	0.041 *	1.2 [0.78–1.85]	0.029	1.23 [0.83–1.85]	0.019 *	1.77 [0.89–1.26]	0.033 *	2.01 [1.03–1.45]	0.032 *	2.03 [0.8–1.27]
	PROMIS Meaning and Purpose in Life	No										
		Somehow	0.046 *	0.87 [0.53–2.1]	0.280	1.4 [0.75–2.63]	0.020 *	2.23 [0.81–1.35]	0.590	1.44 [0.53–0.87]	0.030 *	2.43 [0.51–1.61]
		Yes	0.048 *	0.89 [0.51–2.16]	0.360	1.36 [0.7–2.62]	0.006 **	1.94 [0.68–1.14]	0.430	1.39 [0.47–0.8]	0.040 *	1.92 [0.48–0.86]
R-squared			0.55		0.41		0.62		0.56		0.44	

*p*-value: * *p* < 0.01 ** *p* < 0.01 *** *p* < 0.001. SocMed_MakeDecisions: Using SM information to make decisions about their health. SocMed_DiscussHCP: Using SM information in discussions with their healthcare providers. SocMed_TrueFalse: Finding it hard to tell if SM information is true or false. SocMed_SameViews: People on SM have the same views about health as them. MisleadingHealthInformation: Health-related information on SM being misleading.

## Data Availability

Data is available upon request from the National Cancer Institute.

## Appendix A. Variables of the Study

**Category**	**Code of the Variable**	**Variable**	**Question**	**Responses**
Social media use during the last 12 months	Soc_MedVisited	Visiting social media	In the last 12 months, how often did you visit a social media site?	“Daily,” “sometimes a month,” “less than once a month.”
	SocMed_SharedPers	Using SM to share personal health information	In the last 12 months, how often did you share personal health information on social media?	“Daily,” “sometimes a month,” “less than once a month.”
	SocMed_SharedGen	Using SM to share general health information	In the last 12 months, how often did you share general health-related information on social media (for example, a news article)?	“Daily,” “sometimes a month,” “less than once a month.”
	SocMed_Interacted	Using SM to interact with people with similar health issues	In the last 12 months, how often did you interact with people with similar health or medical issues on social media or online forums?	“Daily,” “sometimes a month,” “less than once a month.”
	SocMed_WatchedVid	Using SM to watch health-related videos	In the last 12 months, how often did you watch a health-related video on a social media site (for example, YouTube)?	“Daily,” “sometimes a month,” “less than once a month.”
Perceptions of social media use for health-related purposes	SocMed_MakeDecisions	Using SM information to make decisions about their health	How much do you agree or disagree—I can use information from social media to make decisions about my health.	“Strongly agree,” “agree,” “somewhat disagree,” and “strongly disagree.”
	SocMed_DiscussHCP	using SM information in discussions with their healthcare providers	How much do you agree or disagree—I can use information from social media in discussions with my healthcare provider.	“Strongly agree,” “agree,” “somewhat disagree,” and “strongly disagree.”
	SocMed_TrueFalse	Finding it hard to tell if SM information is true or false	How much do you agree or disagree—I find it hard to tell whether health information on social media is true or false.	“Strongly agree,” “agree,” “somewhat disagree,” and “strongly disagree.”
	SocMed_SameViews	People on SM have the same views about health as them	How much do you agree or disagree? Most people in my social media networks have the same views about health as I do.	“Strongly agree,” “agree,” “somewhat disagree,” and “strongly disagree.”
	MisleadingHealthInformation	Health-related information on SM being misleading.	How much health information do you see on social media that you think is false or misleading?	“Strongly agree,” “agree,” “somewhat disagree,” and “strongly disagree.”
Personal factors	Age	Age categories	What is your age?	“18–34”,“35–49”,“50–64”,“65 or more”
	Race	Race categories	What is your race?	“White”, “Black”, “Hispanic”, “Other”
	Education	Education categories	What is the highest degree you obtained?	“Some high school or less,” “High school graduate,” “Some college,” “College graduate or more”
	Income	Income categories	What is your yearly income?	“Less than 20K”, “20k to 35k”, “35k to 50k”, “50k to 75k”, “75k or more”
	Gender	Gender categories	What is your gender?	“Female”, “Male”
	general health	Health Perception	In general, how would you describe your health?	“Bad”, “good”, “fair”
	SelfEfficacy	One’s ability to take care of their health	Overall, how confident are you about taking good care of your health?	“Bad”, “good”, “fair”
Environmental factors	FeelIsolated	Feeling isolated	I feel isolated from others	“Yes,” “No,” “Somehow”
	FeelLeftOut	Feeling left out	I feel left out	“Yes,” “No,” “Somehow”
	FeeLPeopleBarelyLKnow	Feel that people barely know them	I feel that people barely know me	“Yes,” “No,” “Somehow”
	FeelPeopleNotWithMe	Feel that people are around but not with them	I feel that people are around me but not with me	“Yes,” “No,” “Somehow”
	PROMIS Social Isolation	Feeling isolated from people	The score of the previous four questions	“Yes,” “No,” “Somehow”
	LifeHasMeaning	Feeling that life has a meaning	My life has meaning	“Yes,” “No,” “Somehow”
	LifeHasPurpose	Feeling that life has a purpose	My life has a purpose	“Yes,” “No,” “Somehow”
	ClearSenseDirection	Having a clear sense of direction in life	I have a clear sense of direction in my life	“Yes,” “No,” “Somehow”
	PROMIS Meaning and Purpose in Life	Having a meaning and purpose to life	The score of the previous questions	“Yes,” “No,” “Somehow”
